# Perceived exertion and regional thermal asymmetry responses following simulated match play in female handball players

**DOI:** 10.3389/fpubh.2026.1935269

**Published:** 2026-10-14

**Authors:** Tevfik Cem Akalın, Nebahat Eler

**Affiliations:** 1 Department of Physical Education and Sports, Faculty of Sports Sciences, Zonguldak Bulent Ecevit University, Zonguldak, Türkiye; 2 Department of Coaching Education, Faculty of Sports Sciences, Gazi University, Ankara, Türkiye

**Keywords:** artificial intelligence, female handball, infrared thermography, match simulation, rating of perceived exertion, thermal asymmetry

## Abstract

**Introduction:**

This study examined changes in lower-extremity thermal asymmetry following simulated match-play in U17 female handball players and evaluated their relationships with global rating of perceived exertion (RPE) and self-reported hand preference. Software-defined thermal alert categories were summarized descriptively.

**Methods:**

A single-group pretest-posttest quasi-experimental design was used. Thirty-five U17 female national-team handball players completed a 50-min match simulation consisting of five 10-min blocks. Bilateral thermal asymmetry in six lower-extremity regions was assessed before the simulation and after a standardized 10-min post-simulation recovery period using infrared thermography and automated region-of-interest segmentation in ai4sports. Global RPE was recorded using the Borg CR10 scale. Holm-adjusted *p* values were calculated within each family of multiple comparisons.

**Results:**

After Holm correction, thermal asymmetry increased significantly in the rectus femoris (mean change = 0.134 °C, adjusted *p* < 0.001, dz = 0.75), vastus group (mean change = 0.163 °C, adjusted *p* = 0.027, dz = 0.49), and gastrocnemius (mean change = 0.246 °C, adjusted *p* = 0.018, dz = 0.53). Changes in the hamstrings, hip adductors, and tibialis anterior were not significant after adjustment. RPE increased from 2.23 ± 0.49 to 6.03 ± 1.25 (*p* < 0.001, Rosenthal r = 0.88; rank-biserial r = 1.00). No statistically detectable association was observed between post-simulation RPE and thermal-asymmetry changes; confidence intervals were compatible with effects in both directions. No hand-preference comparison remained significant after Holm correction. Descriptively, 12 of 35 athletes (34.3%) showed at least one region in the software-defined Caution or Urgent category.

**Conclusion:**

Global perceived exertion increased substantially, while regional thermal-asymmetry responses varied across anatomical regions. No statistically detectable association between RPE and regional thermal changes was observed in this sample, although moderate associations cannot be excluded because of limited precision. Automated thermal image analysis may standardize region-of-interest extraction, but the observed asymmetries and software-defined alert categories should not be interpreted as diagnostic or injury-prediction measures.

## 1 Introduction

Handball is an intermittent team sport characterized by the frequent repetition of high-intensity actions, including acceleration, deceleration, rapid changes of direction, jumping, throwing, and physical contact. Due to these characteristics, the sport imposes significant mechanical and physiological demands during both the offensive and defensive phases (1, 2). Repetitive loading during training and competitive matches can generate a substantial workload, particularly on the lower extremity muscle groups, trunk stabilizers, and the kinetic chain responsible for force transmission to the upper extremities. Specifically, the gluteus maximus and hip adductors are integral to hip stabilization, changes of direction, and propulsive force generation; the quadriceps and hamstring muscle groups facilitate running, jumping, landing, and deceleration; the gastrocnemius-soleus complex and Achilles tendon are responsible for plantar flexion and force production; while the tibialis anterior plays a crucial role in dorsiflexion, ankle control, and neuromuscular regulation during the landing phase (3, 4). Furthermore, the core musculature serves a supportive function in handball-specific throwing and contact maneuvers by contributing to the maintenance of trunk stability and the transfer of force to the upper extremities (5).

Monitoring acute responses to training and competition is important for assessing recovery requirements and managing training loads. Fatigue can be associated with performance decrements and increased recovery needs (6). Various metrics, including jump performance, force outputs, perceived exertion, muscle soreness, and biochemical markers, are used to monitor neuromuscular fatigue in team sports (7). Although MRI and ultrasound provide detailed information for evaluating muscle injuries, their field use is limited by cost and accessibility (8, 9). Infrared thermography (IRT) provides an adjunctive assessment of regional skin-surface temperature and bilateral asymmetry and has been applied in sports monitoring and screening research (10–15). IRT findings should not be interpreted as standalone evidence of muscle damage, inflammation, or injury risk and must be considered alongside environmental conditions, measurement timing, body composition, and individual baselines (16). Automated image-analysis approaches may support more standardized anatomical ROI identification and data extraction (17, 18). RPE is a practical measure of global perceived exertion in team sports (19). Assessing RPE together with regional thermal asymmetry may therefore provide complementary, rather than interchangeable, information about post-match responses.

Infrared thermography has been used in football, basketball, and handball to characterize exercise-related changes in skin-surface temperature and bilateral asymmetry. Lower-extremity injuries are also prominent in handball epidemiology, which has encouraged interest in feasible field-monitoring approaches (20, 21). Studies in handball have mainly examined training sessions, specific exercise protocols, or isolated anatomical regions (22–26). Evidence remains limited in adolescent female players assessed after match-simulated loading, particularly when regional thermal asymmetry is considered alongside global perceived exertion. The present study therefore examined whether these monitoring measures showed statistically detectable associations, while treating automated image analysis as a method for ROI segmentation rather than as a validated injury-prediction system.

Accordingly, the primary objective was to examine lower-extremity thermal asymmetry responses following simulated match-play in U17 female handball players and to evaluate their relationships with global RPE and self-reported hand preference. Software-defined thermal alert categories were summarized descriptively. The following hypotheses were tested:

*H1*: Thermal asymmetry values in the assessed lower-extremity regions differ between pre- and post-simulation measurements.

*H2*: Global RPE scores are higher after the match simulation than before the warm-up and simulation.

*H3*: Post-simulation global RPE is associated with acute changes in lower-extremity thermal asymmetry.

*H4*: Acute changes in lower-extremity thermal asymmetry and RPE differ according to self-reported preferred hand.

In the current study, thermal imaging findings were utilized as a complementary assessment tool for monitoring post-match acute regional skin temperature responses, rather than as a diagnostic criterion for injury or a standalone indicator of muscle damage.

## 2 Methods

### 2.1 Experimental design

This study used a single-group pretest-posttest quasi-experimental design. A handball-specific match simulation was followed by assessment of acute lower-extremity thermal asymmetry and global perceived exertion. Repeated measurements within the same cohort allowed within-athlete pre-post comparisons. Because there was no control group, the results were interpreted as temporal changes observed following the simulation and not as causal effects attributable solely to the protocol.

To standardize the measurements and minimize environmental confounding, all assessments were conducted during the national-team training camp using the same protocol. Athletes rested in the controlled measurement environment for approximately 20 min before baseline imaging. Baseline infrared images and pre-simulation RPE were obtained before a 30-min team warm-up comprising static and dynamic exercises and before the match simulation. The full measurement sequence was: 20-min acclimatization, baseline IRT and RPE, 30-min warm-up, 50-min match simulation, 10-min recovery, and post-simulation IRT and RPE. The assessed regions were the hamstrings, hip adductors, rectus femoris, vastus group, gastrocnemius, and tibialis anterior. Hand preference was determined by athlete self-report as the hand normally used for passing and shooting. This variable was used only as a hand-preference grouping variable and was not interpreted as direct evidence of lower-limb functional dominance. The match simulation lasted 50 min and comprised five 10-min blocks conducted simultaneously on two courts in separate 7-v-7 formats. In each block, 28 athletes played and 7 entered the planned recovery/substitution rotation. The rotation schedule allocated one 10-min recovery block to each athlete, corresponding to a planned active exposure of 40 min per athlete. Actual external load and second-by-second playing time were not recorded.

### 2.2 Participants

Thirty-five female handball players from the U17 Women’s National Handball Team, with a mean age of 16.60 ± 0.65 years (range: 15–17 years), voluntarily participated in the study (Table 1). Participants were recruited purposively because they represented the available national-team cohort that met the study criteria (27).

**Table 1:** Participant characteristics (*N* = 35).

Variable	Mean ± SD	Min	Max
Age (years)	16.60 ± 0.65	15	17
Training age (years)	10.14 ± 1.96	5	15
Height (m)	1.70 ± 0.08	1.51	1.84
Body mass (kg)	67.46 ± 11.48	46.2	94.9
BMI (kg/m^2^)	23.28 ± 3.10	18.04	30.99

BMI, Body Mass Index; SD, Standard deviation.

The inclusion criteria for the participants were established as follows:

- Absence of any acute lower extremity injury or a history of surgical intervention within the preceding six months;
- Provision of written informed consent from a parent or legal guardian and written age-appropriate assent from the athlete.

Given that all participants were aged 17 years or younger, written informed consent was obtained from a parent or legal guardian before study participation. Each athlete received age- and developmentally appropriate information about the study purpose, procedures, and potential implications and provided written age-appropriate assent before participation.

After the match simulation, athletes completed a standardized 10-min recovery period before thermal imaging began. Anterior and posterior lower-extremity images of all 35 athletes were obtained over approximately 5 min. Original image-acquisition timestamps were not retained after transfer to the ai4sports system, which recorded only the upload date; therefore, athlete-level acquisition times and imaging order could not be retrospectively verified or modeled. Post-simulation global RPE was also recorded. Bilateral thermal asymmetry was calculated for each region, and software-defined alert categories were summarized descriptively.

### 2.3 Data collection procedures

#### 2.3.1 Anthropometric measurements

Participants’ stature was measured to the nearest 0.1 cm using a stadiometer (Seca 213, Germany), and body mass was assessed to the nearest 0.1 kg using a digital scale (Tanita BC-418, Japan). All measurements were conducted during the morning hours, with participants barefoot and wearing lightweight sportswear. Body Mass Index (BMI) was calculated by dividing body mass in kilograms by the square of stature in meters (kg/m^2^). These measurements were used to describe the sample. The procedures followed standard anthropometric protocols and posed no risk to the participants.

#### 2.3.2 Infrared thermography (IRT)

Lower-extremity skin-surface temperature was assessed using infrared thermography. Measurement procedures followed the TISEM consensus recommendations for participant preparation, environmental conditions, camera placement, timing, and reporting (28). Thermal images were processed using ai4sports® software (version 3.5.0; AiVisionTech Elektronik Yazılım A.Ş., Türkiye), which automatically segments predefined anatomical regions of interest and exports bilateral temperature-difference values. In this study, the artificial intelligence component was used for automated ROI segmentation and data extraction only. It was not evaluated as a diagnostic or injury-prediction model. Independent reliability and validity of the automated segmentation were not tested in this U17 female cohort. The exported asymmetry measure was a non-negative absolute bilateral temperature difference (ΔT = |left–right|), rather than a signed dominant-minus-nondominant value. The exported dataset did not retain the separate left and right ROI summaries; therefore, whether the proprietary calculation used the ROI mean or maximum temperature could not be independently verified.

In version 3.5.0, the software classified ΔT as Normal (≤0.40 °C), Monitoring (>0.40–0.70 °C), Protective attention (>0.70–1.00 °C), Caution (>1.00–1.50 °C), or Urgent (>1.50 °C). According to the developer documentation supplied to the authors, the 0.40 °C lower boundary was informed by published physiological bilateral-difference ranges, while the higher bands represent a graded software adaptation of previously reported thermographic screening scales (11, 12, 29–31). These thresholds were treated only as software-defined descriptive alerts and were not considered validated diagnostic or injury-risk cutoffs.

Pre- and post-simulation thermal images were captured using a HIKMICRO M30 thermal camera with an infrared resolution of 384 × 288 pixels. Baseline images were obtained after approximately 20 min of rest in the measurement environment and before the 30-min static and dynamic warm-up. Post-simulation infrared imaging was performed following the standardized 10-min recovery period and was completed within approximately 5 min. Imaging was conducted under the same controlled environmental conditions at 20–24 °C, with minimal airflow and stable lighting. Participants had no topical creams, gels, or adhesive tape on the assessed skin. Images were obtained at a distance of 1.0–1.5 m with the camera positioned perpendicular to the skin surface. The assessed regions were selected because handball involves repeated sprinting, jumping, landing, deceleration, and changes of direction (32). The resulting values were interpreted as bilateral skin-temperature asymmetry rather than measures of deep-muscle temperature, tissue damage, or clinical injury.

#### 2.3.3 Rating of perceived exertion (RPE)

The Borg Category-Ratio 10 (CR10) scale was utilized to evaluate the participants’ perceived exertion levels prior to and following the match simulation. The Borg CR10 is a highly practical assessment tool that enables individuals to subjectively quantify the global physical exertion experienced during exercise. Within this scale, a score of 0 denotes “no exertion at all,” whereas a score of 10 corresponds to “maximal exertion” (33).

RPE is a subjective indicator predicated on the individual’s holistic perception of the cardiorespiratory, metabolic, and neuromuscular responses elicited during exercise. RPE-based assessments have been extensively reported as a valid, practical, and cost-effective modality for monitoring exercise intensity, particularly in team sports characterized by variable-intensity and intermittent efforts (19, 34, 35). However, given that session load was not calculated by multiplying the RPE scores by the exercise duration in the current study, the derived values were interpreted strictly as an index of perceived exercise intensity, rather than as a metric of internal training load.

RPE was recorded at two time points. The pre-simulation value was obtained within 5 min before the warm-up and was intended to describe the athletes’ current global perception of exertion or residual fatigue before the protocol; it was not interpreted as exertion produced by the match simulation. The post-simulation rating was obtained within 10 min after play and referred to the athletes’ global perceived exertion during the complete simulation.

The RPE measurements were administered via one-on-one interviews. Participants were visually presented with the Borg CR10 scale, graded from 0 to 10, and each was asked to indicate the numerical value that best reflected their perceived global physical exertion during the match. The scale anchors were explicitly explained to the participants, emphasizing that their responses must be predicated solely on their individual subjective perception. To preclude potential biases or influences from teammates, coaches, and environmental stimuli, the assessments were conducted in a quiet, distraction-free setting. The entire data collection process was executed by the same researchers to ensure methodological consistency.

### 2.4 Statistical analysis

Statistical analyses were performed using SPSS version 26.0 for Windows. All reported statistics were recomputed and verified against the source dataset by the first author (TCA) using the same software. Distributional assumptions for paired thermal change scores were assessed using the Shapiro–Wilk test. Statistical significance was set at *p* < 0.05. To limit family-wise type I error, Holm-adjusted *p* values were calculated separately for the six pre-post thermal comparisons, the six RPE-thermal correlations, and the seven exploratory hand-preference comparisons. Raw and adjusted *p* values are reported. Spearman coefficients are presented with percentile-bootstrap 95% confidence intervals based on 20,000 participant-level resamples. With N = 35, a two-sided alpha of 0.05, and 80% power, a Fisher-z sensitivity calculation indicated an approximate minimum detectable correlation of |rho| = 0.46; smaller associations therefore could not be excluded reliably.

Descriptive statistics included mean, standard deviation, minimum, maximum, frequency, and percentage as appropriate. Post-simulation values were additionally classified using the five software-defined thermal alert categories. Maximum-category distributions and region-level Caution/Urgent occurrences were summarized descriptively; no inferential comparison was performed across these categories because the categories were derived from the same post-simulation thermal values.

#### 2.4.1 Comparative analyses (match effect)

Pre- and post-match comparisons: Paired-samples t tests were used for pre-post thermal asymmetry comparisons. The Wilcoxon signed-rank test was used for pre-post RPE because its distribution did not meet the parametric assumption.

Hand-preference comparisons: Welch’s independent-samples t tests were used to compare thermal change scores and RPE change scores between athletes reporting right- and left-hand preference. Welch’s test was selected because the groups were markedly unequal (30 right, 5 left) and does not require equal variances. These analyses were considered exploratory.

#### 2.4.2 Correlation analyses

Relationship between RPE and thermal asymmetry: Spearman rank correlations were used to examine associations between post-simulation global RPE and the pre-post thermal-asymmetry change score for each region. An exploratory sensitivity analysis repeated these correlations using ΔRPE (post-pre) to align both variables as change scores. Holm adjustment was applied separately to each six-correlation family.

Effect Size Analysis: For paired thermal comparisons, Cohen’s dz. was calculated as the mean paired change divided by the standard deviation of the paired change scores. Percentile-bootstrap 95% confidence intervals were obtained using 20,000 resamples. For the Wilcoxon test, Rosenthal’s r was calculated as |Z|/√N. The matched-pairs rank-biserial correlation was also reported, together with the numbers of positive, negative, and tied differences. Effect sizes were interpreted using Cohen’s conventional thresholds (36).

## 3 Results

After Holm correction, significant increases in thermal asymmetry were retained for the rectus femoris (adjusted *p* < 0.001), vastus group (adjusted *p* = 0.027), and gastrocnemius (adjusted *p* = 0.018). The tibialis anterior increase was significant before adjustment (raw *p* = 0.037) but not after Holm correction (adjusted *p* = 0.112). Hamstrings and hip-adductor changes were not significant (Table 2).

**Table 2:** Pre-post comparisons of lower-extremity thermal asymmetry.

Region	Pre, mean ± SD	Post, mean ± SD	Mean change (95% CI)	t(34)	Raw p	Holm p	dz (95% CI)
Hamstrings	0.249 ± 0.200	0.239 ± 0.183	−0.010 (−0.104, 0.084)	−0.217	0.829	0.829	−0.04 (−0.40, 0.30)
Hip adductors	0.294 ± 0.282	0.404 ± 0.294	0.110 (−0.017, 0.238)	1.766	0.086	0.173	0.30 (−0.03, 0.70)
Rectus femoris	0.168 ± 0.135	0.302 ± 0.198	0.134 (0.073, 0.195)	4.443	<0.001	<0.001	0.75 (0.42, 1.19)
Vastus group	0.317 ± 0.236	0.480 ± 0.298	0.163 (0.048, 0.279)	2.880	0.007	0.027	0.49 (0.17, 0.87)
Gastrocnemius	0.297 ± 0.248	0.543 ± 0.381	0.246 (0.087, 0.405)	3.138	0.004	0.018	0.53 (0.22, 0.90)
Tibialis anterior	0.425 ± 0.321	0.612 ± 0.451	0.188 (0.012, 0.364)	2.165	0.037	0.112	0.37 (0.03, 0.78)

Values are in °C. Mean change was calculated as post-test minus pre-test. Holm p values were adjusted across the six regional comparisons. dz = Cohen’s paired-samples effect size. CI = confidence interval.

Global RPE increased from 2.23 ± 0.49 before the warm-up and simulation to 6.03 ± 1.25 after the simulation (Z = −5.19, *p* < 0.001, Rosenthal r = 0.877; rank-biserial r = 1.00). All 35 athletes had positive changes; there were no negative or tied differences (Table 3).

**Table 3:** Pre-post comparison of global RPE.

Measurement	Pre, mean ± SD	Post, mean ± SD	Median change	Z	*p*	Rosenthal r; rank-biserial r
Global RPE	2.23 ± 0.49	6.03 ± 1.25	4.00	−5.19	<0.001	0.877; 1.00

RPE = rating of perceived exertion. Rosenthal’s r was calculated as |Z|/√N. All 35 paired differences were positive, with no negative or tied differences; the matched-pairs rank-biserial correlation was therefore 1.00. The Z value includes correction for tied absolute ranks.

No statistically detectable association was observed between post-simulation RPE and any regional thermal-asymmetry change score. All 95% confidence intervals included zero and were sufficiently wide to include potentially meaningful associations in at least one direction. Holm adjustment did not change this interpretation (Table 4).

**Table 4:** Spearman correlations between post-simulation global RPE and thermal asymmetry changes.

Region	Spearman rho	95% CI	Raw p	Holm p
Hamstrings	−0.215	(−0.488, 0.099)	0.215	1.000
Hip adductors	−0.220	(−0.557, 0.144)	0.203	1.000
Rectus femoris	−0.036	(−0.373, 0.299)	0.836	1.000
Vastus group	0.243	(−0.109, 0.569)	0.159	0.956
Gastrocnemius	−0.068	(−0.391, 0.262)	0.697	1.000
Tibialis anterior	−0.162	(−0.477, 0.187)	0.353	1.000

In the exploratory ΔRPE sensitivity analysis, no correlation with regional thermal change was statistically significant after Holm adjustment: hamstrings rho = −0.211 (raw *p* = 0.223, adjusted *p* = 1.000), hip adductors rho = −0.206 (raw *p* = 0.236, adjusted *p* = 1.000), rectus femoris rho = 0.016 (raw *p* = 0.929, adjusted *p* = 1.000), vastus group rho = 0.305 (raw *p* = 0.075, adjusted *p* = 0.451), gastrocnemius rho = −0.024 (raw *p* = 0.893, adjusted *p* = 1.000), and tibialis anterior rho = −0.057 (raw *p* = 0.746, adjusted *p* = 1.000).

No thermal or RPE change score differed significantly according to self-reported hand preference after Holm correction. The gastrocnemius comparison yielded raw *p* = 0.023, but the Holm-adjusted value was *p* = 0.164 and was not interpreted as statistically significant (Table 5).

**Table 5:** Exploratory comparisons according to self-reported hand preference.

Outcome	Right-hand preference mean ± SD	Left-hand preference mean ± SD	Welch t(df)	Raw p	Holm p
Hamstrings change	0.012 ± 0.277	−0.141 ± 0.234	1.318 (6.05)	0.235	1.000
Hip-adductor change	0.163 ± 0.342	−0.206 ± 0.410	1.907 (4.97)	0.115	0.691
Rectus femoris change	0.131 ± 0.190	0.153 ± 0.083	−0.432 (12.61)	0.673	1.000
Vastus-group change	0.154 ± 0.355	0.219 ± 0.200	−0.591 (8.97)	0.569	1.000
Gastrocnemius change	0.298 ± 0.473	−0.069 ± 0.241	2.662 (10.22)	0.023	0.164
Tibialis-anterior change	0.193 ± 0.493	0.157 ± 0.684	0.114 (4.72)	0.914	1.000
RPE change	3.700 ± 1.343	4.400 ± 1.342	−1.080 (5.43)	0.326	1.000

Thermal outcomes and RPE are pre-post change scores. Welch’s t tests were used because the hand-preference groups were markedly unequal (30 right, 5 left). Thermal changes are in °C. Holm *p* values were adjusted across the seven exploratory comparisons. Hand preference was self-reported and does not represent lower-limb functional dominance.

The maximum post-simulation alert category was Normal for 4 athletes (11.4%), Monitoring for 7 (20.0%), Protective attention for 12 (34.3%), Caution for 11 (31.4%), and Urgent for 1 (2.9%). These categories are descriptive software outputs and do not indicate validated injury-risk levels (Table 6).

**Table 6:** Distribution of maximum post-simulation software-defined thermal alert categories.

Maximum alert category	Threshold	*n*	%
Normal	≤0.40 °C	4	11.4
Monitoring	>0.40–0.70 °C	7	20.0
Protective attention	>0.70–1.00 °C	12	34.3
Caution	>1.00–1.50 °C	11	31.4
Urgent	>1.50 °C	1	2.9

Seventeen region-level Caution or Urgent occurrences were identified. Eleven occurred in the tibialis anterior (64.7%), four in the gastrocnemius (23.5%), and two in the vastus group (11.8%). No Caution or Urgent occurrence was identified in the hamstrings, hip adductors, or rectus femoris (Table 7).

**Table 7:** Regional distribution of Caution and Urgent thermal alert occurrences.

Region	Caution/Urgent occurrences, n	% of 17 occurrences
Tibialis anterior	11	64.7
Gastrocnemius	4	23.5
Vastus group	2	11.8
Hamstrings	0	0.0
Hip adductors	0	0.0
Rectus femoris	0	0.0

Twelve of the 35 athletes (34.3%) had at least one region classified in the Caution or Urgent category (Table 8).

**Table 8:** Summary of Caution or Urgent thermal alert occurrence.

Summary statistic	Value
Total Caution/Urgent region-level occurrences	17
Total athletes	35
Athletes with ≥1 Caution/Urgent region	12 (34.3%)

Effect-size estimates indicated a large standardized paired change for rectus femoris, moderate changes for the vastus group and gastrocnemius, and a large RPE response. Although tibialis anterior had a small-to-moderate effect estimate, its Holm-adjusted *p* value was not significant. Effect sizes were interpreted together with their confidence intervals and adjusted *p* values (Table 9).

**Table 9:** Summary of effect-size estimates for pre-post outcomes.

Outcome	Test	Statistic	Adjusted p	Effect size (95% CI where applicable)
Global RPE	Wilcoxon	Z = -5.19	<0.001	Rosenthal r = 0.877; rank-biserial r = 1.00
Hamstrings	Paired t	t (34)= − 0.22	0.829	dz = −0.04 (−0.40, 0.30)
Hip adductors	Paired t	t (34)=1.77	0.173	dz = 0.30 (−0.03, 0.70)
Rectus femoris	Paired t	t (34)=4.44	<0.001	dz = 0.75 (0.42, 1.19)
Vastus group	Paired t	t (34)=2.88	0.027	dz = 0.49 (0.17, 0.87)
Gastrocnemius	Paired t	t (34)=3.14	0.018	dz = 0.53 (0.22, 0.90)
Tibialis anterior	Paired t	t (34)=2.17	0.112	dz = 0.37 (0.03, 0.78)

For thermal outcomes, p values are Holm-adjusted across six comparisons and effect sizes are Cohen’s dz with bootstrap 95% CIs. RPE was evaluated with the Wilcoxon signed-rank test and Rosenthal’s r.

## 4 Discussion

This study examined acute lower-extremity thermal-asymmetry responses following simulated match-play in U17 female handball players. After correction for multiple comparisons, significant increases were retained for the rectus femoris, vastus group, and gastrocnemius, whereas changes in the hamstrings, hip adductors, and tibialis anterior were not statistically significant. Global RPE increased markedly. No statistically detectable association between RPE and regional thermal changes was observed, but the confidence intervals and sample-size sensitivity indicate that moderate associations cannot be excluded. No hand-preference comparison remained significant after multiplicity correction. These findings suggest that global perceived exertion and regional bilateral thermal asymmetry provide non-equivalent information within this protocol, without establishing that they are independent or dissociated constructs.

The increases retained for the rectus femoris, vastus group, and gastrocnemius may reflect region-specific bilateral responses to repeated jumping, acceleration, deceleration, landing, and change-of-direction actions. Physical demands in female handball vary by playing position, including total distance, high-intensity running, accelerations, decelerations, and player load (37). However, these mechanisms were not measured directly in the present study, and the observed thermal changes should therefore not be attributed to specific actions or mechanical loads with certainty.

Thermal asymmetry should not be interpreted as a direct indicator of muscle activity, muscle damage, or injury. Infrared thermography quantifies skin surface temperature rather than deep muscle tissue and is influenced by cutaneous blood flow, sweating, environmental conditions, camera setup, imaging timing, prior activity, and body composition (28, 38). Therefore, the observed increases were interpreted as acute regional skin-temperature asymmetry following, rather than caused by, the match simulation.

The absence of significant alterations within the hamstrings and hip adductors does not imply that these regions were not subjected to mechanical loading during the match. Thermal asymmetry inherently reflects the temperature differential between the bilateral sides. Consequently, symmetrical temperature changes occurring across both extremities may result in low asymmetry values, even when the total absolute load is substantial. Correspondingly, Lino-Samaniego et al. (2022) reported that following isometric and auxotonic squat protocols in male handball players, lower extremity skin temperature initially decreased and approached baseline levels within approximately 15 min; however, no significant temperature discrepancy manifested between the right and left sides. This finding underscores the necessity of evaluating absolute skin temperature alterations and bilateral thermal asymmetry as distinct physiological variables (25).

Post-exercise thermal responses are time dependent. Masur et al. (39) reported that skin-temperature-related parameters commonly decrease during the first 15 min after exercise and may subsequently increase, whereas Pinheiro et al. (40) reported increased temperatures in several lower-extremity regions approximately 10 min after a sprint protocol in elite female soccer players. In the present study, imaging began 10 min after the simulation and was completed within approximately 5 min. Although athlete order within this 10–15-min interval was not included as an analytical covariate, it may still have contributed to variability.

The pronounced elevation of RPE from 2.23 to 6.03 indicates that the simulation was perceived as substantial physical exertion. No statistically detectable correlation was observed between post-simulation RPE and changes in thermal asymmetry, and the exploratory analysis using ΔRPE produced the same inferential conclusion. However, with *N* = 35 the study had approximately 80% power only for correlations of about |rho| ≥ 0.46. The confidence intervals therefore remain compatible with smaller or moderate associations. RPE represents a global perceptual response influenced by cardiorespiratory strain, muscle fatigue, central motor command, psychological state, and prior recovery, whereas thermal asymmetry represents a bilateral temperature difference within a specific anatomical region (41). The present results should therefore be interpreted as a lack of a detectable association in this sample, not as evidence that no relationship exists.

The present finding aligns with the results of de Carvalho et al. (42), who reported no significant correlation between skin temperature and perceived fatigue, recovery status, muscle soreness, or creatine kinase levels in professional soccer players. Similarly, in professional padel players, although thermal discrepancies were observed in specific regions post-training, it was indicated that these alterations were not associated with changes in global fatigue and muscle soreness (43). Conversely, Majano et al. (44) demonstrated that specific regional thermal asymmetries in elite soccer players could be correlated with stress, muscle soreness, fatigue, and RPE. However, the varying directionality of these correlations across different anatomical regions renders it challenging to postulate a generalized and linear relationship between RPE and thermal asymmetry. Furthermore, Masur et al. (39) concluded that the associations between IRT parameters and other internal load metrics remain inconsistent.

The exploratory hand-preference analysis did not identify any statistically significant group difference after Holm correction. The gastrocnemius comparison had a raw *p* value of 0.023, but this finding was not retained after Holm correction (adjusted *p* = 0.164). Moreover, preferred throwing hand cannot be assumed to represent lower-limb functional dominance during jumping, landing, or change-of-direction tasks. The pronounced group imbalance (30 right-hand and 5 left-hand athletes) further limits precision. Accordingly, these findings do not support a hand-preference-specific thermal response.

Software-defined alert categories were retained only for descriptive reporting. Twelve athletes had at least one Caution or Urgent region, most frequently involving the tibialis anterior. These counts identify where larger bilateral differences occurred in this dataset, but they do not establish clinical severity or injury probability. Inferential comparisons across categories were not performed because both the grouping variable and the regional outcomes were derived from the same post-simulation thermal measurements, creating circularity.

Artificial intelligence-supported image analysis can facilitate automated ROI segmentation and standardized data extraction. In the present study, ai4sports was used for these functions only. The study did not test segmentation agreement against manual expert annotation, test–retest reliability, external validity in adolescent female athletes, or injury-prediction performance. Therefore, the findings support the feasibility of automated processing within this protocol, not the clinical validity of the software or its alert thresholds.

## 5 Conclusion

Following Holm correction, thermal asymmetry increased significantly in the rectus femoris, vastus group, and gastrocnemius, while global RPE increased substantially. No statistically detectable association between RPE and regional thermal changes was observed in this sample, although the confidence intervals do not exclude moderate associations. Automated thermal analysis may complement athlete monitoring by facilitating standardized regional ROI processing; however, neither a single asymmetry value nor a software-defined alert category should be interpreted as evidence of injury, muscle damage, or future injury risk.

## 6 Practical applications

RPE and thermal imaging may provide complementary information. RPE summarizes global perceived exertion, whereas thermal imaging describes regional bilateral skin-temperature differences. Training restrictions or clinical decisions should not be based on a single thermal measurement or fixed software threshold. Thermal findings should be considered together with individual baselines, symptoms, prior injury, neuromuscular performance, and external-load measures (39, 45).

## 7 Limitations

The single-group pretest-posttest design and use of simulated rather than official match-play limit causal interpretation. Baseline IRT and RPE were recorded before the 30-min warm-up; therefore, the pre-post changes reflect the combined influence of the warm-up and the 50-min match simulation rather than the simulation alone. The sample was restricted to 35 U17 female national-team players, limiting generalizability. Although the protocol scheduled four 10-min active blocks per athlete, actual second-by-second playing time, positional exposure, accelerations, decelerations, jumps, contacts, and external load were not recorded. The hand-preference groups were also markedly unequal, and preferred throwing hand did not directly measure lower-limb functional dominance.

Post-simulation imaging began after a standardized 10-min recovery period and was completed over approximately 5 min. However, original image-acquisition timestamps were not retained after transfer to the analysis system, so athlete-level acquisition time and order could not be verified or modeled. Rapid time-dependent changes in skin temperature may therefore have contributed to variability. The correlation analyses were also imprecise: with *N* = 35, the approximate minimum detectable correlation at 80% power was |rho| = 0.46, and the confidence intervals did not exclude smaller or moderate associations. Because asymmetry was expressed as an absolute bilateral difference, low-baseline regions may also be susceptible to floor effects and regression toward the mean. Menstrual-cycle phase, hydration, prior training load, sleep, and body composition were not controlled analytically. The dataset retained only exported bilateral ΔT values rather than separate left and right ROI temperature summaries, and the study did not independently evaluate the reliability or validity of ai4sports automated ROI segmentation in this population. The software-defined alert thresholds were not validated here as injury-risk categories. Future studies should retain image timestamps and raw ROI summaries, use repeated standardized imaging times, objective external-load monitoring, functional lower-limb dominance measures, and longitudinal clinical outcomes.

## Data Availability

The original contributions presented in the study are included in the article/supplementary material, further inquiries can be directed to the corresponding author.
